# COVID19 vaccine intentions in South Africa: health communication strategy to address vaccine hesitancy

**DOI:** 10.1186/s12889-021-12196-4

**Published:** 2021-11-17

**Authors:** Umakrishnan Kollamparambil, Adeola Oyenubi, Chijioke Nwosu

**Affiliations:** 1 University of the Witwatersrand, Johannesburg, South Africa; 2 University of the Free State, Bloemfontein, Human Sciences Research Council, Cape Town, South Africa

**Keywords:** Vaccine hesitancy, Health behaviour models, risk perception, Efficacy, COVID19, South Africa

## Abstract

**Background:**

Vaccine hesitancy is emerging as a significant challenge in many parts of the world in the fight against the COVID19 pandemic. The continued infection amongst the unvaccinated can lead to a heightened risk of further virus mutation, exposing even those vaccinated to new virus strains. Therefore, there are social benefits in minimising vaccine hesitancy. The objective of this study is to assess the level of COVID19 vaccine hesitancy in South Africa, identify the socio-economic patterns in vaccine hesitancy and highlight insights from the national survey that can inform the development of a COVID-19 vaccination acceptance communication campaign.

**Methods:**

The study uses the nationally representative National Income Dynamics Study - Coronavirus Rapid Mobile Survey (NIDS-CRAM) survey. The analysis combines univariate and bivariate statistics, as well as multivariate regression models like binomial/ordinal and multinomial logit.

**Results:**

The study finds that vaccine acceptance is lower than that of non-pharmaceutical intervention like face-mask use. Only 55% fully accept the vaccine, while a further 16% are moderately accepting of vaccines. Together, vaccine acceptance is estimated at 70.8%, and vaccine hesitancy against COVID19 is estimated at 29.2% amongst the adult South African population.

The study has identified the perceived risk of infection with the mediating role of efficacy as a key predictor of vaccine intention. Higher awareness of COVID19 related information and higher household income are correlated with lower vaccine hesitancy. The non-black African population group has significantly high vaccine hesitancy compared to black Africans.

**Conclusions:**

There are other significant differences across socio-economic and demographic variables in vaccine hesitancy. From a communication perspective, it is imperative to continue risk messaging, hand in hand with clearer information on the efficacy of the vaccines.

**Supplementary Information:**

The online version contains supplementary material available at 10.1186/s12889-021-12196-4.

## Background

While the focus of protective behaviour for the first year of the COVID19 pandemic relied on non-pharmaceutical interventions (NPI) like the use of face-mask, hand hygiene, social distancing, and staying at home; the successful development of various COVID19 vaccines has expanded the available approaches to protective behaviour in South Africa. Previous studies have shown that acceptance of NPI based protective behaviour is high in South Africa and has improved from 92% in May–June 2020 to 97% in July–August 2021 as the pandemic progressed [1, 2]. However, the concerns regarding side-effects of vaccines is much higher compared to NPI. Therefore the behavioural response to both can differ substantially. Hence, it is time to assess the vaccine intentions of the population to put in place measures to improve vaccine acceptance.

The focus of the government has now shifted to making vaccines available and its roll-out strategy. It is equally important to ensure the public uptake of vaccines once they become widely available [3]. The success of the vaccination programme depends upon achieving a sufficient scale to result in community immunity. Further, continued infection amongst the unvaccinated can lead to a heightened risk of further virus mutation, exposing even those vaccinated (against the earlier strains of the virus) to new virus strains [4]. Therefore, there are social benefits in minimising vaccine hesitancy.

Pre-COVID19 studies have acknowledged that behavioural response against vaccination can be specific to a particular vaccine depending on the perceived risk of infection and the confidence in the vaccine [5]. Sallam (2021) [6] has highlighted the wide variation between COVID19 vaccine acceptance between countries. Therefore, it is highly relevant to assess vaccine intentions in South Africa regarding the COVID vaccine and explore ways to improve vaccine acceptance in the general public health interest.

Various studies have shown that health communication has an important role in influencing individual responses to health risks [7–9]. Hence, it is imperative to develop a health communication strategy to encourage individuals to accept both NPI and vaccination against COVID19.

While different theories and models have been put forth by health communication theorists designed for health messaging, the Health Belief [10] and the Extended Parallel Process (EPP) [11, 12] models are suited in the pandemic context because of their foundation on health risk [13, 14]. Therefore, it is important to understand the constructs of risk and efficacy (or the belief that the health response can yield the desired effect) to encourage protective behaviour against COVID19 [15, 16].

The Health Belief Model (HBM) is one of the earliest health models to use key variables such as risk susceptibility/vulnerability, risk severity, efficacy, and barriers to behavioural change perception, to predict health response behaviour [10]. EPPM builds on this foundation by also using the tenets of the parallel process model (PPM) [17], which distinguished between two independent reactions to fear appeals, viz., (a) a cognitive response of risk management process, leading to protective behaviour, and (b) an emotional response of fear management process, that leads to denial, and avoidance. However, PPM did not explain the contexts when either of these responses is evoked. EPPM extends the PPM to incorporate the four elements of Protection Motivation Theory (PMT) (the perceived severity of a threatening event, the perceived vulnerability, the response efficacy of the recommended preventive behaviour, and the perceived self-efficacy) [18] to explain the contexts when protective behaviour or avoidance response is evoked.

The extended parallel process model (EPPM) posits that efficacy plays a mediating role in the relationship between risk perception and response to the fear appeal message [11, 12]. Based on the interaction between risk perception and perceived efficacy, Witte (1992) identifies two possible outcomes even with high perceived risk (High risk-High efficacy and High risk-Low efficacy) based on the available risk reduction measures. One of the implications of this is recognition that greater fear does not necessarily lead to greater message acceptance, but can perversely cause message rejection, a possibility not acknowledged by HBM. Moreover, according to EPPM, if the individual perceives low risk, there is no motivation to adopt behavioural change even if the individuals have high efficacy. For instance, in the context of the COVID-19 pandemic, the High risk-Low efficacy category of the population would benefit from health communication that focuses on the efficacy and benefits of vaccination as they are already aware of the health risks COVID-19. The population category that perceives the low risk of the pandemic (irrespective of their efficacy perception) would need to be educated about the pandemic related health risks first. Communication to the High risk-High efficacy category needs to focus on calls to vaccination with information on vaccination access. Therefore, identifying the risk-efficacy interaction is relevant in tailoring the communication strategy to improve health response behaviour [19–25].

Studies within the COVID context are fast emerging using the EPPM framework [26–29] or the HBM framework [30]. However, these studies relate primarily to non-vaccine behaviour. Exceptions are Chu (2021) [31] in the US context and Lin et al. (2020) [28] in the context of China, which used the EPPM and HBM frameworks, respectively, to assess COVID19 vaccine behaviour. Edwards et al. (2021) [32] has identified the correlates of vaccine hesitancy in Australia without using a specific theoretical framework. The authors have not been able to identify a study in the South African context on COVID19 vaccine intention.

This study aims to assess the level of COVID19 vaccine hesitancy in South Africa, identify the socio-economic patterns in vaccine hesitancy, and develop insights from the national survey that can inform the development of a COVID-19 vaccination acceptance communication campaign.

## Data & Methods

### Data

The study primarily uses the fourth wave of the National Income Dynamics Study - Coronavirus Rapid Mobile Survey (NIDS-CRAM 2021) survey [33]. The NIDS-CRAM survey is a special follow up with a subsample of adults from households in Wave 5 of the National Income Dynamics Study (NIDS) run by the Southern Africa Labour and Development Research Unit (SALDRU). The NIDS is South Africa’s first nationally representative household-level panel survey undertaken approximately in two-year intervals between 2008 and 2017, covering a wide range of socio-economic, health, labour and household-related information. The NIDS-CRAM survey is designed to be nationally representative. Despite the smaller sample size (compared to NIDS), it remains the best available source of quantitative information on a national scale to assess the socio-economic impact of the coronavirus pandemic in South Africa. More detailed technical information on NIDS-CRAM surveys is provided by Ingle et al. (2021), Kerr et al. (2020), Daniels et al. (2020) and Branson and Wittenberg 2019 [34–37].

The fourth wave of the NIDS-CRAM has a sample covering complete questionnaire information for 5629 individuals. The fourth wave of the NIDS-CRAM survey was conducted over February (96%) and March (4%) 2021. Therefore, the questions were administered under adjusted alert level 3 (Feb 2–28 Feb 2021, when curfew restrictions were imposed and businesses had to adhere to regulations on the maximum number of persons on their premises) and adjusted alert level 1 (March 2021, when most normal activities were allowed to resume with precautions and health guidelines being followed) lockdown conditions.

Wave 4 of NIDS-CRAM introduced a question on vaccine intention, and hence this research is based on it.

Table 1 provides the description of variables used in the analysis. Apart from the key risk perception variable, the study uses age and pre-existing chronic illness of the respondent to control for severity of risk [38, 39]. The household income variable is an important variable to control for the feasibility of behavioural change. The survey response for this variable, though, has a high proportion of missing information. As such, rand value responses to household income variables have been supplemented with the median value of the income bracket responses. This approach can, however, distort the income distribution. Therefore, following Wittenberg (2017) [40], we reweight those who provide rand amounts using the inverse probability that an individual will report a rand amount in that bracket. Further, alternate models are estimated where socio-economic status is controlled by including other variables such as the recent experience of hunger in the household, the presence of grant recipients, and electrified dwelling as additional checks.

**Table 1:** Variable definitions

Variable	Instrument question	Response
Vaccine intention	‘To what extent do you agree or disagree with the statement: If a vaccine for COVID-19 were available, I would get it?’	strongly agree/ somewhat agree/ somewhat disagree/strongly disagree
Risk of infection	‘Do you think you are likely to get the coronavirus?’	Yes/No
Efficacy	‘Can you avoid getting Coronavirus?’, ‘Vaccine ineffectiveness is not the main reason why you would not take a vaccine for COVID-19’. To create a vaccine-specific efficacy variable, the two questions are interacted. If the respondent does not state vaccines to be ineffective, it takes the value one and believes that coronavirus can be avoided.	Yes/No
Age	Age of the individual	years
Chronic illness	Do you have any of these chronic conditions (you don’t have to tell us which one): HIV, TB, lung condition, heart condition or diabetes?”.	Yes/No
Covid Awareness	a binary variable is constructed which takes the value 1 if the respondent is aware of the three most important symptoms of infection (cough, breathlessness and fever).	Binary
Education	Number of years of education completed	years
Socio-economic status	Per capita Household income	Rands
Hunger	In the last 7 days has anyone in your household gone hungry because there wasn’t enough food? Yes = 1, No = 1	Binary
Grant receiving household	The household received at least one form of social grant = 1, 0 otherwise	Binary
Electricity	Dwelling with electricity = 1, 0 otherwise	Binary
Sex:	Male = 1, 0 otherwise	Binary
Race:	black African = 1, 0 otherwise	Binary
Religious:	Religion is important = 1, 0 otherwise	Binary
Employed	Employed = 1, 0 otherwise	Binary
Partnered	Married or living with partner = 1, 0 otherwise	Binary
NPI behaviour	The number of non-pharmaceutical measure adopted to prevent COVID infection	0–8

Because individual responses emerge from a complex interaction of different social, cultural, political and personal factors in vaccine decision [41], additional socio-economic variables such as sex (male), race (black African), partnered, location (urban) and religiosity are incorporated to get a clearer picture of the range of possible predictors about vaccine intention.

### Methods

We estimate the concentration index of vaccine hesitancy, risk perception and efficacy variables to analyse the socio-economic inequality in the distribution of vaccine intention and COVID risk-efficacy perceptions in South Africa. The socio-economic criteria considered are household per capita income, age and educational outcomes of all individuals in the sample. Since vaccine hesitancy, risk and efficacy are binary variables, the Erreygers-corrected concentration index (Erreygers 2009) is calculated as:

$$ E(h)=\frac{4\mu }{b_h-{a}_h}\ast \left(\ \frac{2}{n\mu}{\sum}_{i=1}^n{h}_i{r}_i-1\right) $$

where n is sample size, h is the binary vaccine hesitancy variable, bh and ah are the maximum (1) and minimum (0) of the binary variable, μ its mean and r the rank of individual i by income from poorest to richest. E(h) is expected to lie between + 1 and − 1, with a positive value indicating that vaccine hesitancy is distributed more among the higher end of the income distribution and a negative value indicating that it is concentrated more among the lower end of the income distribution (Kollamparambil 2020).

The study uses logit regression to model the probability of vaccine hesitancy to identify its predictors. Two theoretical models are used for regression specification; a) Model A with the two target variables of risk perception and efficacy specified as separate binary variables and b) Model B with the inclusion of the interaction of risk perception and efficacy variables in the form of High risk-Low efficacy (denial) and Low-risk groups. Both the denial and low-risk groups are benchmarked in the model against the responsive (High risk-High efficacy) group. The logit estimation of Models 1 and 2 are specified as follows:

1 $$ Vac\_{Hes}_i={\beta}_0+{\beta}_1\left( Risk\_{perception}_i\right)+{\beta}_2\left({Efficacy}_i\right)+\gamma {X}_i+{\epsilon}_i $$

2 $$ Vac\_{Hes}_i={\beta}_0+{\beta}_1\left({Denial}_i\right)+{\beta}_2\left( Low\_{risk}_i\right)+\gamma {X}_i+{\epsilon}_i $$

Where,

*Vac* _ *Hes*_*i*_ denotes the vaccine intention for individual i, taking the value 1 for hesitancy and 0 otherwise,

*Risk* _ *perception* is a binary variable where 1 is assigned to individuals who perceive the risk of COVID19 infection and 0 otherwise,

*Efficacy* is a binary variable where 1 is assigned to individuals who perceive vaccine efficiency and the possibility to avoid COVID19 infection and 0 otherwise,

*Denial* is a binary variable where 1 is assigned to individuals with high-risk perception and low efficacy perception.

*Low* _ *risk* is a binary variable where 1 is assigned to individuals with low efficacy perception.

Xi denotes a vector of explanatory variables such as age, sex, race, education, household income, COVID awareness, religiosity, socio-economic status etc. that characterise individual i,

ϵi encapsulates the error term.

The predicted probability of vaccine hesitancy based on Models A and B are estimated as:

$$ Prob\left( Vac\_{Hes}_i\right)=\frac{1}{1+{\mathit{\exp}}^{-{Z}_i}} $$

Following identifying the predictors of vaccine hesitancy using logit regress, the study undertakes the multinomial regression to identify the differentiating characteristics of responsive, denial and low-risk groups. The responsive category is the benchmark regression for estimation. Therefore the multinomial logistic regression used to predict categorical placement within denial and low-risk groups are specified as follows:

$$ {Denial}_i={\propto}_0+{\propto}_1{X}_i+{\theta}_i $$

$$ Low\_{risk}_i={\lambda}_0+{\lambda}_1{X}_i+{\theta}_i $$

The vector X of independent variables include age, race, sex, education, employment status, marital status, socio-economic status proxies, regional dummy variables etc. The regression error term is denoted by *θ*.

## Results

The descriptive statistics of the key socio-economic characteristics of the sample for the multivariate regression indicate resonance with national statistics (Table 2).

**Table 2:** Summary statistics of the Study Sample

Variable	Obs	Mean	Std. Dev.	Min	Max
Age, years	4440	40.47	15.04	18	90
18–35	4440	0.274	0.446	0	1
36–60	4440	0.617	0.486	0	1
Above 60	4440	0.108	0.310		
Chronic illness	4440	0.168	0.374	0	1
Covid Awareness	4440	0.096	0.295	0	1
Household per capita income, Rands	4440	2854	6135	0	150,000
Education, years	4440	11.52	3.729	0	22
African	4440	0.782	0.413	0	1
Male	4440	0.482	0.500	0	1
Married/with partner	4440	0.479	0.500	0	1
Urban	4440	0.760	0.427	0	1
Electricity	4440	0.946	0.226	0	1
Experienced Hunger	4440	0.164	0.370	0	1
Employed	4440	0.497	0.500	0	1
Social Grant recipient	4440	0.353	0.478	0	1
Religious	4440	0.913	0.282	0	1

Source: NIDS-CRAM weighted. # All variables except age, education and income are binary

### Preliminary analysis

**Fig. 1:**
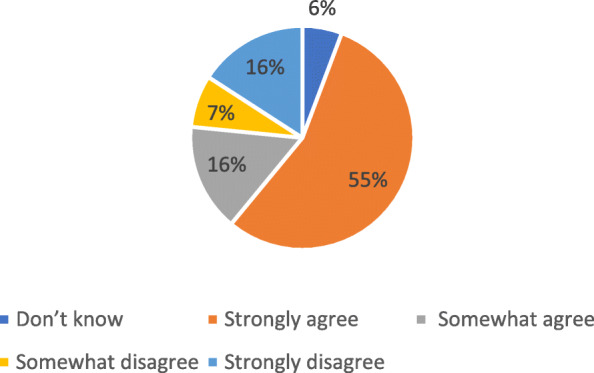
If a vaccine for COVID-19 were available, I would get it?. Source: NIDS-CRAM wave 4. *n* = 5613, weighted

Our analysis of vaccine intention indicates that 55% of the population have a strong acceptance of the vaccine, followed by 16% who have a lower certainty to accept the vaccine (Fig. 1). Stronger uncertainty is expressed by 7% who somewhat reject the vaccine and a further 16% that strongly rejected the vaccine. For purposes of this analysis we include the 6% who did not respond to the question as being vaccine hesitant.

Vaccine acceptance is defined as those who ‘strongly agree’ or ‘somewhat agree’ while those who ‘strongly disagree’ with ‘somewhat disagree’ and ‘don’t know’ are included in the vaccine hesitant category. Vaccine hesitancy is calculated as 29.16% (with a 95% confidence interval of 28–30%).

**Fig. 2:**
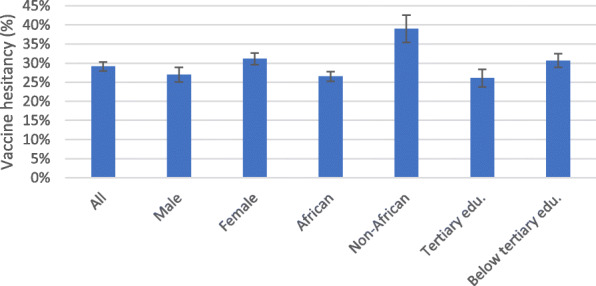
Vaccine hesitancy, Sex, Race and Education. Source: NIDS-CRAM wave 4, weighted

Vaccine hesitancy across sex, race and education attainment is presented in Fig. 2. A difference across sexes is significant at 95% confidence level, with females in the sample having higher level vaccine hesitancy (31%) compared to males (27%). Further, the difference across population groups is signficant at 99% confidence level, with the non-black African population reporting higher hesitancy (39%), compared to the black African population (26%). Tertiary education is another differentiating factor, with those with tertiary education recording significantly lower hesitancy (26%) than those with below tertiary education (31%).

**Fig. 3:**
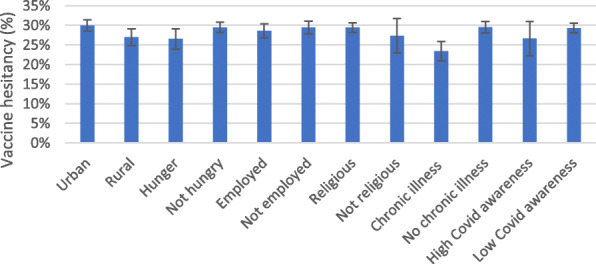
Vaccine hesitancy and key characteristics. Source: NIDS-CRAM wave 4, weighted

Geographical location is found to be also a significant differentiating factor, with vaccine hesitancy found to be higher in urban areas (30%) compared to rural areas (27%) (Fig. 3). Individuals who reported chronic illness have on an average lower (23%) vaccine hesitancy compared to those who did not report chronic illness (30%). While these findings are relevant and point to possible predictors, some variables may have confounding factor-like income. For instance, poverty and hunger are higher in rural areas in South Africa. Thus, it is important to undertake a multivariate analysis before drawing firm conclusions on the predictors of vaccine hesitancy.

**Fig. 4:**
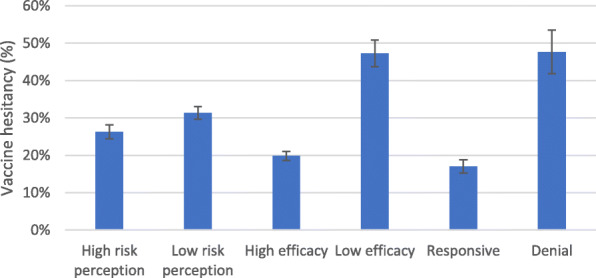
Vaccine hesitancy, Risk and Efficacy. Source: NIDS-CRAM wave 4, weighted

Figure 4 highlights a significant difference between the vaccine hesitancy rates between those who reported high risk perception and those who did not with the former catefory having signficanty lower hesitancy (26%) compared to the latter (31%) (Fig. 4). Similarly, those who reported higher self-efficacy had significantly lower vaccine hesitancy in relation to those without self-efficiacy. Further statistically significant differences are evident in the hesitancy across the efficacy groups and the risk-efficacy interaction groups (responsive and denial) with the responsive group having the lowest vaccine hesitancy (17%).

Table 3 uses the concentration index to assess the socio-economic inequality in the prevalence of vaccine hesitancy and the risk and efficacy parameters along with the income, age, and education distributions. As vaccine hesitancy, risk perception and efficacy variables are binary, the Erreygers-corrected concentration index [42] is used to assess their socio-economic inequality.

**Table 3:** Erreygers concentration index of vaccine hesitancy, risk and efficacy

CI	Vaccine hesitancy	Risk	Efficacy	Responsive (High risk-high efficacy)	Denial (High risk-Low efficacy)	Low risk
Income	−0.017	0.223***	−0.03	0.143***	0.043***	−0.223***
	(0.031)	(0.035)	(0.024)	(0.030)	(0.016)	(0.35)
Age	−0.121***	0.111***	−0.003	0.023	−0.014	− 0.111***
	(0.023)	(0.026)	(0.017)	(0.023)	(0.009)	(0.026)
Education	−0.018	0.132***	0.045***	0.114***	0.029**	−0.133***
	(0.023)	(0.027)	(0.018)	(0.025)	(0.011)	(0.027)

Standard errors in parentheses, *** *p* < 0.01, ** *p* < 0.05, * *p* < 0.1

While income-based inequality is not evident for vaccine hesitancy, it is clear that income plays a key role in risk and efficacy (Table 3). Vaccine hesitancy is negative and significant along with age, pointing to higher acceptance among the elderly. The higher hesitancy concentration among the young could be related to the perceived lower severity of the infection-related morbidity and mortality among the younger age groups [43, 44].

Risk perception is significantly higher among those with higher income. Similar results have been reported by Kollamparambil and Oyenubi (2021) [30] based on waves 1 and 2 of the NIDS-CRAM. Our results show that this pattern is sustained after a year. As expected, risk perception has a significant positive concentration along with the age distribution, indicating higher risk perception among the older individuals (Table 3). This is not surprising considering the available information that age increases the severity of COVID illness, the risk of hospitalisation and a higher COVID infection-related mortality rate [38, 39]. This is related to the results observed regarding vaccine hesitancy and points to the mediating role of risk perception in leading to the higher vaccine hesitancy among younger age groups.

Both risk perception and efficacy are found to be pro-education. The risk-efficacy interaction variables are led by risk in terms of their concentration patterns. ‘Responsive’ (high risk-high efficacy) and ‘Denial’ (high risk-low efficacy) are both pro-rich, aligned with the results of risk perception. The low-risk category (combination of low-risk-high efficacy and low risk-low efficacy groups) are pro-poor. Low-risk is also pro-young and pro-low education.

The findings indicate that although vaccine hesitancy is not concentrated along with the income or education distribution, they can nevertheless play a role through mediating factors like risk perception, which have definite concentration along with income, age and education distributions.

### Multivariate analysis

This section undertakes two sets of multivariate analysis: first, to identify the correlates of vaccine hesitancy with particular focus on identifying the role of risk and efficacy variables (Table 4); and second, to identify the characteristics of the population groups that comprise the high risk-high efficacy (responsive), high risk-low efficacy (denial) and low-risk groups (Table 5).

**Table 4:** Marginal effects: Logit regression Dependent variable, Vaccine Hesitancy

VARIABLES	(1)	(2)	(3)	(4)	(5)	(6)
Risk perception	−0.056**	− 0.064**	− 0.057**			
	(−0.103 - -0.009)	(−0.117 - -0.011)	(− 0.104 - -0.009)			
Efficacy	−0.258***	−0.275***	− 0.251***			
	(−0.334 - -0.183)	(−0.357 - -0.192)	(− 0.327 - -0.175)			
Low risk^a^				0.085***	0.092***	0.081***
				(0.036–0.135)	(0.039–0.145)	(0.032–0.131)
Denial^a^				0.262***	0.268***	0.256***
				(0.145–0.379)	(0.150–0.387)	(0.138–0.375)
Age above 60 years@	− 0.128***	− 0.150***	− 0.141***	− 0.125***	− 0.140***	− 0.141***
	(− 0.184 - -0.072)	(− 0.217 - -0.083)	(− 0.194 - -0.087)	(− 0.190 - -0.059)	(− 0.211 - -0.068)	(− 0.204 - -0.078)
Age 31–60 years@	− 0.065**	− 0.073**	− 0.066**	− 0.069**	− 0.073**	− 0.079***
	(− 0.119 - -0.010)	(− 0.134 - -0.012)	(− 0.120 - -0.011)	(− 0.129 - -0.009)	(− 0.137 - -0.009)	(− 0.136 - -0.021)
Chronic illness	− 0.028	− 0.031	− 0.028	− 0.049*	− 0.052*	− 0.046*
	(−0.083–0.027)	(−0.091–0.030)	(−0.083–0.027)	(−0.103–0.004)	(−0.108–0.005)	(−0.100–0.008)
COVID Information	−0.038	− 0.044	− 0.045	− 0.060*	− 0.066*	− 0.064*
	(− 0.102–0.025)	(−0.115–0.027)	(− 0.108–0.018)	(−0.128–0.008)	(− 0.138–0.006)	(− 0.131–0.003)
Education	−0.007**	− 0.010**	− 0.009***	− 0.008**	− 0.010**	− 0.010***
	(−0.014 - -0.001)	(− 0.019 - -0.001)	(− 0.015 - -0.002)	(− 0.015 - -0.002)	(− 0.019 - -0.002)	(− 0.017 - -0.004)
log PC HH Income	− 0.016**			− 0.016**		
	(−0.030 - -0.001)			(−0.032 - -0.001)		
2. PC HH Income _Q2		0.005			−0.002	
		(−0.065–0.075)			(− 0.073–0.068)	
3. PC HH Income _Q3		− 0.059*			−0.052	
		(−0.119–0.000)			(− 0.118–0.014)	
4. PC HH Income _Q4		− 0.035			−0.033	
		(− 0.099–0.030)			(− 0.102–0.035)	
5. PC HH Income _Q5		− 0.032			−0.030	
		(−0.106–0.042)			(− 0.108–0.048)	
Hunger			−0.009			−0.010
			(−0.067–0.050)			(− 0.069–0.048)
Electricity			0.002			−0.028
			(−0.094–0.099)			(− 0.137–0.082)
Grant receiving Household			0.034			0.027
			(−0.016–0.084)			(− 0.022–0.076)
African	− 0.159***	− 0.162***	− 0.154***	− 0.144***	− 0.139***	− 0.139***
	(− 0.252 - -0.066)	(− 0.265 - -0.059)	(− 0.246 - -0.061)	(− 0.234 - -0.053)	(− 0.235 - -0.044)	(− 0.228 - -0.049)
NPI behaviour	− 0.022**	− 0.025*	− 0.023**	− 0.030***	− 0.032**	−0.031***
	(−0.044 - -0.001)	(−0.053–0.003)	(−0.044 - -0.001)	(− 0.051 - -0.009)	(− 0.059 - -0.006)	(− 0.052 - -0.010)
Male	− 0.031	− 0.039	− 0.034	− 0.030	− 0.035	−0.039*
	(− 0.076–0.014)	(− 0.089–0.012)	(−0.079–0.010)	(−0.077–0.017)	(− 0.086–0.015)	(−0.084–0.006)
Married/Partnered	0.030	0.030	0.034	0.021	0.019	0.029
	(−0.017–0.077)	(−0.021–0.082)	(−0.013–0.081)	(−0.028–0.071)	(−0.033–0.071)	(−0.020–0.077)
Employed	0.049*	0.050*	0.041	0.023	0.018	0.018
	(−0.000–0.098)	(−0.008–0.108)	(−0.008–0.090)	(−0.029–0.075)	(−0.039–0.074)	(−0.031–0.068)
Religious	−0.028	− 0.034	−0.033	− 0.024	−0.026	− 0.034
	(−0.124–0.069)	(−0.138–0.071)	(−0.130–0.064)	(−0.120–0.072)	(−0.126–0.073)	(−0.130–0.061)
Province controls	yes	yes	yes	yes	yes	yes
Wald chi2	138.57**	158.31***	158.14***	115.46***	122.23***	115.96***
Observations	3642	3642	3611	3950	3950	3912

Robust ci in parentheses, *** *p* < 0.01, ** *p* < 0.05, * *p* < 0.1

^a^the benchmark category is Responsive (High risk-high efficacy), @ the benchmark category is 18–30 years

**Table 5:** Multinomial logit regression: Base regression, Responsive category

VARIABLES	Denial^a^	Low risk^a^
Age, above 60 yrs.@	− 0.801*	0.178
	(−1.727–0.125)	(− 0.297–0.654)
Age, 31–60 yrs.@	− 0.528*	− 0.457***
	(−1.121–0.066)	(− 0.751 - -0.163)
Education, yrs	− 0.004	− 0.042**
	(− 0.082–0.075)	(− 0.078 - -0.005)
African	− 0.870**	0.158
	(− 1.580 - -0.159)	(− 0.303–0.619)
COVID Information	0.055	− 0.383**
	(−0.563–0.673)	(−0.760 - -0.006)
Chronic illness	−0.240	−0.303**
	(−0.785–0.304)	(−0.596 - -0.011)
Household Income per capita (log)	0.002	−0.104**
	(−0.175–0.180)	(−0.198 - -0.011)
Receiving government grant	0.365	−0.196
	(−0.155–0.885)	(−0.448–0.056)
Employed	−0.211	− 0.672***
	(−0.756–0.335)	(−0.951 - -0.393)
Male	0.263	−0.028
	(−0.217–0.743)	(−0.272–0.217)
Married/Partnered	0.100	− 0.334***
	(−0.378–0.578)	(−0.586 - -0.081)
Urban	0.068	−0.038
	(−0.616–0.751)	(−0.300–0.224)
Electricity	−0.114	− 0.271
	(−1.484–1.255)	(−0.807–0.266)
Religious	0.998*	− 0.325
	(−0.165–2.161)	(−0.801–0.152)
Province control	yes	yes
Constant	−1.849	2.991***
	(−4.668–0.971)	(1.806–4.176)
Wald chi2(46)	206.46***	206.46***
Observations	3665	3665

Robust ci in parentheses, *** *p* < 0.01, ** *p* < 0.05, * *p* < 0.1

^a^Base regression: Responsive (High risk-high efficacy), @ Base category Age 18–30 years

Models 1–3 in Table 4 include risk and efficacy as separate binary variables, whereas models 4–6 include risk-efficacy interaction variables (denial and low-risk categories) in line with the EPPM model. Various specifications are estimated using the log of per capita income, income quintiles and non-financial proxies for socio-economic status (hunger in household, household with electricity, grant recipient in household).

The marginal effects of logit regressions in models 1–3 highlight the role of risk and efficacy in driving vaccine hesitancy while controlling for other pertinent predictors (Table 4). The predicted probability of vaccine hesitancy (model 1) changes by − 0.056 (95%, CI -0.103 -0.009) for an individual who perceives the risk of COVID19 infection. Similarly, the incremental risk associated with the self-efficacy variable on vaccine hesitancy is − 0.258 (99%, CI -0.334 -0.183).

The results of Models 4–6 provide additional insight into the mediating role of the efficacy in determining the effect of risk perception on vaccine hesitancy. The positive marginal effects of the low-risk variable in model 4 indicate that individuals who perceive lower vulnerability to infection are more likely to be vaccine-hesitant [0.085, 99%, CI 0.036 0.135] than the responsive (high risk and high efficacy) group. In addition, the denial (high risk and low efficacy) group have, on average, a higher vaccine hesitancy [0.262, 99%, CI 0.145–0.379] compared to the responsive (high risk and high efficacy) group.

Age categories have a significant negative association with vaccine hesitancy across all estimations, compared against the benchmark age group of 18–31 years. The predicted probability of vaccine hesitancy is lowest amongst the oldest age group of above sixty years [Model 1: − 0.128, 99%, CI -0.184 -0.072], while the age group 31 to 60 years also had a negative marginal effect [Model 1: -0.065, 95% CI -0.119 -0.010].

Chronic illness is also significant in the models with interacted risk-efficacy variables [Model 4: -0.049, 90% CI -0.103 - 0.004]. Education is associated with lower vaccine hesitancy [Model 4: − 0.008, 95%, CI -0.015 -0.002].

The negative association between the adoption of NPI behaviour and vaccine hesitancy is evident across all models. In model 4, the marginal effects of NPI indicate a reduction in the predicted probability of vaccine hesitancy by − 0.030 [99%, CI -0.051 -0.009]. There are other interesting socio-economic predictors of vaccine intention revealed through the estimations. The black African individuals have significantly lower vaccine hesitancy [Model 4: − 0.144, 99%, CI -0.234 -0.053] compared to other race groups.

In order to address any potential bias caused by the construct of the efficacy variable, the observations where only vaccine hesitancy drives the efficacy variable are excluded in a restricted estimation. In other words, in order to ensure that no definitional relationship runs between the efficacy variable and the dependent variable of vaccine hesitancy, observations where vaccine ineffectiveness takes the value one, and the general efficacy variable is one are excluded from the estimation.. The results from this restricted estimation are given in the appendix (Table A1) and validate Table 4.

As a further robustness check to the logit regression (Table 4), we retain the response to the vaccine intention on a four-point scale to estimate a ordinal logit regressions (Table A2). The results that risk perception, through the mediation of efficacy, is driving vaccine intentions are consistent with the logit regression in Table 4.

The multivariate analysis of predictors of vaccine hesitancy has identified denial and low-risk perception (low risk-low efficacy and low risk- high efficacy) categories as positive predictors of vaccine hesitancy. Therefore, it is important to target these groups for health communication based on health-risk appeal and efficacy messaging. This requires identifying the socio-economic and demographic characteristics of these groups. For this purpose, the study next undertakes a multinomial logit regression, where the base category is the responsive (high risk-high efficacy) category (Table 5). It must be flagged here that the construct of the risk categories is based on risk perception and not the severity of the risk.

A non-linear relationship between age and the low-risk perception group is indicated by the results. The age group, 30–60, are less likely to belong to the low-risk group [− 0.457, 99%, CI -0.751 -0.163] compared to the benchmark category (responsive group) (Table 5). However, those above 60 years are not significantly different across the risk-efficacy groups. On the other hand, education decreases the probability [− 0.042, 95%, CI -0.078 -0.005] of belonging to the low-risk perception group, vis-à-vis the responsive group. Being married [− 0.334, 99%, CI -0.586 -0.081] and being employed [− 0.672, 99%, CI -0.951 -0.393] have a similar effect of reducing the probability of being in the low-risk perception group. Per capita, household income also decreases the probability [− 0.104, 95%, CI -0.198 -0.011] of being in the low-risk perception group.

There are not many significant identifiers of the denial group (high risk-low efficacy) from the responsive group (high risk-high efficacy). The black African individuals have a lower probability [− 0.870, 95%, CI -1.580 -0.159] of being in the denial category than the responsive category. Albeit at a 90% confidence level, those who consider religion important are more likely to be in the denial category.

## Discussion

Based on the findings of this study and the meta-analysis by Sallam (2021) [6], we find that vaccine hesitancy in South Africa is lower than in countries like Kuwait, Jordan, Italy, Russia, Poland, US, and France; but higher than other countries like Ecuador, Malaysia, Indonesia and China.

The study has found that the health behaviour models offer important predictors of vaccine intentions. Risk perception (both vulnerability and severity) is a key driving factor along with efficacy. Further, the results indicate that efficacy plays a mediating role in the relationship between risk perception and vaccine behaviour. This study validates the argument by Witte (1992) [11] and Witte et al. (2002) [23] that efficacy plays an important mediating role in the relationship between risk perception and the associated health behavioural response.

The group that was seen to be most accepting of vaccines is those that perceive high risk and high efficacy (responsive group). Compared to responsive individuals, low-risk individuals are more likely to be hesitant regarding the vaccine. We also see a significant difference between the vaccine intention of the ‘responsive’ and the ‘denial’ groups. This points to risk perception as the driving factor, with the clear role of efficacy as the mediating factor. Similarly, the negative association between COVID awareness and vaccine hesitancy points to the need for creating more awareness and education around vaccines to reduce hesitancy. From a communication perspective, the findings highlight the need to continue risk messaging, hand in hand with clearer information on the efficacy of the vaccines.

A plausible explanation for the negative association between household income and vaccine hesitancy is that income improves access and reduces the barriers to vaccines. The other proxies included controlling for socio-economic statuses like the experience of hunger, social grant receipt, and electrified dwelling. However, do not give any strong indications on their role in driving vaccine hesitancy. However, there is still the possibility that socio-economic status acts via risk perception and efficacy.

Our study finds that the lowest risk from COVID19 is perceived by the young, the less informed, low-income individuals, and those without partners (Table 6). Therefore the emerging recommendation is that these groups are targeted for health-risk appeal, which focusses on the vulnerability to and severity of COVID19 infection. Enhanced risk perception is expected to improve vaccine acceptance amongst these groups of individuals. Our study has highlighted the importance of information on COVID19 in driving the behavioural response to it. Therefore, strong health communication to educate and create awareness on the pandemic will improve vaccine acceptance as well. However, it needs to be emphasised that excessive fear messaging without efficacy-related messaging can lead to mental health issues [45]. Therefore, efficacy messaging cannot be ignored in the health communication strategy. The efficacy related communication needs to be especially targeted to the non-black Africans and the religious individuals who need to be educated on COVID19 vaccine as a credible solution to the coronavirus pandemic. A recommendation emerging from the study is to use religious leaders and institutions for efficacy messaging. This is based on the finding that 91% of South Africans consider religion important and religious individuals are found more likely to belong to the denial (high risk-low efficacy) group. The inputs from this study for the COVID-19 vaccination acceptance communication campaign are summarised in Table 6.

**Table 6:** Key findings for the COVID-19 vaccination acceptance communication campaign

Responsive: People are educated about risk, believe in the efficacy and respond by taking protective action. 40% of population are responsive. Vaccine hesitancy is 26%. Profile: Older, educated, married, well-informed Communication focus: Provide calls to vaccination and make vaccination available. Provide clear information on COVID19 vaccine side effects and effectiveness	Denial: People are aware of the risks but feel helpless to respond effectively. 7% of population fall in this category. Vaccine hesitancy is 27%. Profile: Non-black African, religious Communication focus: Less focus on health-risk messaging and more on protective danger control through educating about solutions Provide clear information on COVID19 vaccine side effects and effectiveness
Low risk: Due to the low-risk perception, people do not enter into efficacy assessment. 52.6% of population fall in this category Vaccine hesitancy is highest at 31%. Profile: Young, low education, single status, less informed, low income Communication focus: Educate through health-risk messaging, risk and protective danger control through awareness about solutions. Provide clear information on COVID19 vaccine side effects and effectiveness	

## Conclusion

The study has highlighted the mediating role of efficacy in the relationship between risk perception and health behaviour relating to vaccine intention. This result is insightful and suggests that it is important to accompany risk messaging with efficacy related communication to improve vaccine acceptance.

Further insights from the study that can inform the development of a COVID-19 vaccination acceptance communication campaign are the low-risk perception group as the young, less educated, less informed, lower-income individuals who can benefit through health-risk messaging. Efficacy messaging needs to focus on non-black African and religious individuals who comprise the denial group. There is an over-arching need to provide research-based information on the side effects and effectiveness of COVID 19 vaccines across the board. The study acknowledges the need for further focussed research that can yield recommendations on the nature of health-risk appeal as well as vaccine-related information that would lead to improved vaccine acceptance.

## Supplementary Information

**Additional file 1.**

## Data Availability

The data is available from http://www.nids.uct.ac.za/nids-cram/data-access The data is publically available for use. All methods were carried out in accordance with relevant guidelines and regulations.

## References

[1] Kollamparambil U, Oyenubi A. Socio-economic inequality in the response to COVID19 pandemic, Policy paper. Cape Town: NIDS-CRAM Wave 2; 2020.

[2] Burger R, Christian C, Maughan-Brown B, Rensburg R, Rossouw L. COVID-19 risk perception, knowledge and behaviour. Cape Town: National Income Dynamics Study (NIDS)–Coronavirus Rapid Mobile Survey (CRAM); 2020.

[3] Dror AA, Eisenbach N, Taiber S, Morozov NG, Mizrachi M, Zigron A, Srouji S, Sela E (2020). Vaccine hesitancy: the next challenge in the fight against COVID-19. Eur J Epidemiol.

[4] Rubin R (2021). COVID-19 vaccines vs variants—determining how much immunity is enough. JAMA.

[5] Dubé E, Laberge C, Guay M, Bramadat P, Roy R, Bettinger JA (2013). Vaccine hesitancy: an overview. Hum Vaccin Immunother.

[6] Sallam M (2021). COVID-19 vaccine hesitancy worldwide: a concise systematic review of vaccine acceptance rates. Vaccines.

[7] Latimer AE, Brawley LR, Bassett RL (2010). A systematic review of three approaches for constructing physical activity messages: what messages work and what improvements are needed?. Int J Behav Nutr Phys Act.

[8] Lustria MLA, Noar SM, Cortese J, Van Stee SK, Glueckauf RL, Lee J (2013). “A meta-analysis of web-delivered tailored health behavior change interventions”: corrigendum. J Health Commun.

[9] Noar SM, Grant Harrington N, Van Stee SK, Shemanski AR (2011). Tailored health communication to change lifestyle behaviors. Am J Lifestyle Med.

[10] Rosenstock IM (1974). Historical origins of the health belief model. Health Educ Monogr.

[11] Witte K (1992). Putting the fear back into fear appeals: the extended parallel process model. Commun Monogr.

[12] Witte K (1994). Fear control and danger control: a test of the extended parallel process model (EPPM). Commun Monogr.

[13] Noar SM (2004). A health Educator’s guide to theories of health behavior. Int Q Community Health Educ..

[14] Dillard JP (1994). Rethinkin the study of fear appeals: an emotional perspective. Commun Theory.

[15] Boster FJ, Mongeau P (1984). Fear-arousing persuasive messages. Ann Int Commun Assoc.

[16] Witte K, Allen M (2000). A meta-analysis of fear appeals: implications for effective public health campaigns. Health Educ Behav.

[17] Leventhal H (1970). Findings and theory in the study of fear communications. Adv Exp Soc Psychol.

[18] Rogers RW (1975). A protection motivation theory of fear appeals and attitude change1. J Psychol.

[19] Carcioppolo N, Jensen JD, Wilson SR, Collins WB, Carrion M, Linnemeier G (2013). Examining HPV threat-to-efficacy ratios in the extended parallel process model. Health Commun.

[20] McKay DL, Berkowitz JM, Blumberg JB, Goldberg JP (2004). Communicating cardiovascular disease risk due to elevated homocysteine levels: using the EPPM to develop print materials. Health Educ Behav.

[21] Slonim AB, Roberto AJ, Downing CR, Adams IF, Fasano NJ, Davis-Satterla L, Miller MA (2005). Adolescents’ knowledge, beliefs, and behaviors regarding hepatitis B: insights and implications for programs targeting vaccine-preventable diseases. J Adolesc Health.

[22] Witte K (1997). Preventing teen pregnancy through persuasive communications: realities, myths, and the hard-fact truths. J Community Health.

[23] Witte K, Girma B, Girgre A (2002). Addressing underlying mechanisms to HIV/AIDS preventive behaviors in Ethiopia. Int Q Community Health Educ.

[24] Barnett DJ, Errett NA, Rutkow L (2013). A threat-and efficacy-based framework to understand confidence in vaccines among the public health workforce. Vaccines.

[25] Smith PJ, Humiston SG, Marcuse EK, Zhao Z, Dorell CG, Howes C (2011). Parental Delay or Refusal of Vaccine Doses, Childhood Vaccination Coverage at 24 Months of Age, and the Health Belief Model. Public Health Rep.

[26] Lithopoulos A, Liu S, Zhang C-Q, Rhodes RE (2021). Predicting physical distancing in the context of COVID-19: a test of the extended parallel process model among Canadian adults. Can Psychol.

[27] Shirahmadi S, Seyedzadeh-Sabounchi S, Khazaei S, Bashirian S, Miresmæili AF, Bayat Z, Houshmand B, Semyari H, Barati M, Jenabi E, Heidarian F, Zareian S, Kheirandish M, Dadae N (2020). Fear control and danger control amid COVID-19 dental crisis: application of the extended parallel process model. PLoS One.

[28] Lin Y, Hu Z, Zhao Q, Alias H, Danaee M, Wong LP (2020). Understanding COVID-19 vaccine demand and hesitancy: a nationwide online survey in China. PLoS Negl Trop Dis.

[29] Yang J, Wu X, Sasaki K, Yamada Y (2020). Changing health compliance through message repetition based on the extended parallel process model in the COVID-19 pandemic. PeerJ.

[30] Kollamparambil U, Oyenubi A (2021). Behavioural response to the Covid-19 pandemic in South Africa.

[31] Chu H, Liu S (2021). Integrating health behavior theories to predict American’s intention to receive a COVID-19 vaccine. Patient Educ Couns.

[32] Edwards B, Biddle N, Gray M, Sollis K (2021). COVID-19 vaccine hesitancy and resistance: correlates in a nationally representative longitudinal survey of the Australian population. PLoS One.

[33] SALDRU. National Income Dynamics Study-Coronavirus Rapid Mobile Survey (NIDS-CRAM), Wave 4 [dataset]. Version Beta3. Cape Town: Allan Gray Orbis Foundation [funding agency]. Cape Town: Southern Africa Labour and Development Research Unit [implementer], 2021. Cape Town: DataFirst [distributor], 2021. 2021. https://cramsurvey.org/reports/.

[34] Ingle K, Brophy T, Daniels RC. National Income Dynamics Study–Coronavirus Rapid Mobile Survey (NIDS-CRAM) panel user manual. Technical Note Version 2020;1.

[35] Kerr A, Ardington C, Burger R (2020). Sample design and weighting in the NIDS-CRAM survey.

[36] Daniels RC, Ingle K, Brophy T (2020). Determinants of attrition in NIDS-CRAM waves 1 & 2.

[37] Branson N, Wittenberg M. Longitudinal and cross-sectional weights in the NIDS data 1–5 [NIDS technical paper number 9]. Cape Town: Southern Africa Labour and Development Research Unit, University of Cape Town; 2019.

[38] Gesesew HA, Koye DN, Fetene DM, Woldegiorgis M, Kinfu Y, Geleto AB, Melaku YA, Mohammed H, Alene KA, Awoke MA, Birhanu MM, Gebremedhin AT, Gelaw YA, Shifti DM, Muluneh MD, Tegegne TK, Abrha S, Aregay AF, Ayalew MB, Gebre AK, Gebremariam KT, Gebremedhin T, Gebremichael L, Leshargie CT, Kibret GD, Meazaw MW, Mekonnen AB, Tekle DY, Tesema AG, Tesfay FH, Tesfaye W, Wubishet BL, Dachew BA, Adane AA (2021). Risk factors for COVID-19 infection, disease severity and related deaths in Africa: a systematic review. BMJ Open.

[39] Pijls BG, Jolani S, Atherley A, Derckx RT, Dijkstra JIR, Franssen GHL, Hendriks S, Richters A, Venemans-Jellema A, Zalpuri S, Zeegers MP (2021). Demographic risk factors for COVID-19 infection, severity, ICU admission and death: a meta-analysis of 59 studies. BMJ Open.

[40] Wittenberg M (2017). Wages and wage inequality in South Africa 1994–2011: part 1 – wage measurement and trends. S Afr J Econ.

[41] Callaghan T, Moghtaderi A, Lueck JA, Hotez P, Strych U, Dor A, et al. Correlates and disparities of intention to vaccinate against COVID-19. Soc SciMed. 2021:113638. 10.1016/j.socscimed.2020.113638. 10.1016/j.socscimed.2020.113638 PMC7834845 33414032

[42] Erreygers G (2009). Correcting the concentration index. J Health Econ.

[43] Bruine de Bruin W (2021). Age differences in COVID-19 risk perceptions and mental health: Evidence from a national US survey conducted in March 2020. J Gerontol B Psychol Sci Soc Sci.

[44] Niño M, Harris C, Drawve G, Fitzpatrick KM (2021). Race and ethnicity, gender, and age on perceived threats and fear of COVID-19: evidence from two national data sources. SSM Popul Health.

[45] Oyenubi A, Kollamparambil U, Nwosu CO (2021). Flip side of risk perception: on the negative influence of risk perception on subjective health during the pandemic.

