# Supplementary material for: COVID19 vaccine intentions in South Africa: health communication strategy to address vaccine hesitancy
Source: BMC Public Health. 2021 Nov 17;21:2113. doi: 10.1186/s12889-021-12196-4 (PMC8596859; doi:10.1186/s12889-021-12196-4)
Supplement: Supplementary file 1 — Additional file 1. [file 12889_2021_12196_MOESM1_ESM.docx]

Appendix

Table A1: Restricted sample logit estimation: Vaccine Hesitancy

|  | (1) | (2) |
| --- | --- | --- |
| VARIABLES |  |  |
|  |  |  |
| Risk perception | -0.342** |  |
|  | (0.146) |  |
| Efficacy | -1.232*** |  |
|  | (0.173) |  |
| Low risk |  | 0.451*** |
|  |  | (0.135) |
| Denial |  | 1.155*** |
|  |  | (0.251) |
| Age | -0.684*** | -0.497** |
|  | (0.239) | (0.204) |
| Chronic illness | -0.233 | -0.319** |
|  | (0.179) | (0.155) |
| Covid information | -0.279 | -0.385* |
|  | (0.225) | (0.210) |
| Log HHincome per capita | -0.0974** | -0.0883** |
|  | (0.0449) | (0.0406) |
| Education | -0.0375** | -0.0359** |
|  | (0.0191) | (0.0173) |
| African | -0.846*** | -0.689*** |
|  | (0.223) | (0.205) |
| NPI behaviour | -0.133** | -0.156*** |
|  | (0.0655) | (0.0564) |
| Male | -0.138 | -0.119 |
|  | (0.138) | (0.125) |
| Married | 0.0441 | -0.0108 |
|  | (0.141) | (0.125) |
| Employed | 0.251* | 0.0862 |
|  | (0.151) | (0.136) |
| Religious | -0.119 | -0.0826 |
|  | (0.282) | (0.249) |
| Constant | 2.228*** | 0.819 |
|  | (0.558) | (0.521) |
| Wald chi2 | 128.66*** | 99.28*** |
| Observations | 3,628 | 3,936 |

**Standard errors in parentheses, *** p<0.01, ** p<0.05, * p<0.1**

Table A2 Ordinal logit, marginal effects: Dependent variable, Vaccine Intention.

| Model1 | High Risk | High Efficacy | age | Chronic illness | Education | Income | information |
| --- | --- | --- | --- | --- | --- | --- | --- |
| Strongly accept vaccine | 0.050** | 0.197*** | 0.007*** | 0.070** | 0.003 | 0.016 | 0.047 |
|  | (0.024) | (0.031) | (0.001) | (0.032) | (0.003) | (0.011) | (0.045) |
| Somewhat accept vaccine | -0.012** | -0.048*** | -0.002*** | -0.017** | -0.001 | -0.004 | -0.011 |
|  | (0.005) | (0.008) | (0.000) | (0.008) | (0.001) | (0.003) | (0.011) |
| Somewhat reject vaccine | -0.009** | -0.038*** | -0.001*** | -0.014** | -0.001 | -0.003 | -0.010 |
|  | (0.004) | (0.009) | (0.000) | (0.006) | (0.001) | (0.002) | (0.009) |
| Strongly reject vaccine | -0.028** | -0.111*** | -0.004*** | -0.039** | -0.002 | -0.009 | -0.026 |
|  | (0.016) | (0.024) | (0.001) | (0.020) | (0.002) | (0.006) | (0.025) |
| Model 2 | Denial ^ | Low risk^ | age | Chronic illness | Education | income | information |
| Strongly accept vaccine | -0.173*** | -0.066*** | 0.007*** | 0.055 | 0.008** | 0.016 | 0.039 |
|  | (0.044) | (0.024) | (0.001) | (0.036) | (0.002) | (0.011) | (0.044) |
| Somewhat accept vaccine | 0.040*** | 0.016*** | -0.002*** | -0.013 | -0.002** | -0.004 | -0.010 |
|  | (0.011) | (0.005) | (0.000) | (0.009) | (0.000) | (0.003) | (0.011) |
| Somewhat reject vaccine | 0.035*** | 0.013*** | -0.002*** | -0.012 | -0.002** | -0.003 | -0.008 |
|  | (0.003) | (0.006) | (0.000) | (0.008) | (0.000) | (0.002) | (0.009) |
| Strongly reject vaccine | 0.037*** | 0.098*** | -0.004*** | -0.030 | -0.005** | -0.009 | -0.022 |
|  | (0.018) | (0.025) | (0.001) | (0.020) | (0.000) | (0.006) | (0.024) |

Robust standard errors in parentheses, *** p<0.01, ** p<0.05, * p<0.1, ^Benchmark category is responsive (high risk-high efficacy)
